# Towards a Synthetic Chloroplast

**DOI:** 10.1371/journal.pone.0018877

**Published:** 2011-04-20

**Authors:** Christina M. Agapakis, Henrike Niederholtmeyer, Ramil R. Noche, Tami D. Lieberman, Sean G. Megason, Jeffrey C. Way, Pamela A. Silver

**Affiliations:** 1 Department of Systems Biology, Harvard Medical School, Boston, Massachusetts, United States of America; 2 Wyss Institute for Biologically Inspired Engineering, Harvard University, Boston, Massachusetts, United States of America; University of California Merced, United States of America

## Abstract

**Background:**

The evolution of eukaryotic cells is widely agreed to have proceeded through a series of endosymbiotic events between larger cells and proteobacteria or cyanobacteria, leading to the formation of mitochondria or chloroplasts, respectively. Engineered endosymbiotic relationships between different species of cells are a valuable tool for synthetic biology, where engineered pathways based on two species could take advantage of the unique abilities of each mutualistic partner.

**Results:**

We explored the possibility of using the photosynthetic bacterium *Synechococcus elongatus* PCC 7942 as a platform for studying evolutionary dynamics and for designing two-species synthetic biological systems. We observed that the cyanobacteria were relatively harmless to eukaryotic host cells compared to *Escherichia coli* when injected into the embryos of zebrafish, *Danio rerio*, or taken up by mammalian macrophages. In addition, when engineered with invasin from *Yersinia pestis* and listeriolysin O from *Listeria monocytogenes*, *S. elongatus* was able to invade cultured mammalian cells and divide inside macrophages.

**Conclusion:**

Our results show that it is possible to engineer photosynthetic bacteria to invade the cytoplasm of mammalian cells for further engineering and applications in synthetic biology. Engineered invasive but non-pathogenic or immunogenic photosynthetic bacteria have great potential as synthetic biological devices.

## Introduction

While the evolution of cooperation and altruism are often seen as paradoxical events in the course of natural selection, endosymbiosis has been recognized as a driver of evolutionary change. Not only has gene exchange been observed between hosts and symbionts [1], but the development of communities suitable to new ecological niches [2] and even the origin of the eukaryotic kingdom hinge on symbiotic collaborations [3], [4]. Modern endosymbiotic relationships between bacteria and eukaryotic organisms reflect a remarkable diversity in how widely disparate species can interact in positive ways, from nutritional symbiosis between *Buchnera* and aphids [5], to nitrogen fixation by *Rhizobia* in plant root nodules [6] and photosynthetic symbiosis between algal chloroplasts and sea slugs [7].

Cooperative behavior and symbiotic relationships are widespread in nature and have recently begun to be exploited in synthetic biological networks of increasing complexity [8]. Multi-component synthetic-ecological systems have been developed for hydrogen production through metabolic engineering [9] and for the production of other useful metabolites [10]. Communication between cells has also been engineered for multiple applications, including pattern formation [11] and oscillators [12]. Engineered communities have also been useful as a generalized model of cooperation and competition in microbial populations [13], [14] and two-species metabolic modeling has been used in the identification of cooperating variants of *E. coli* [15]. While invasive bacteria have been explored as tools for synthetic biology and targeted tumor killing bacteria [16], neutral or beneficial endosymbiosis has not been pursued.

**Figure 1:**
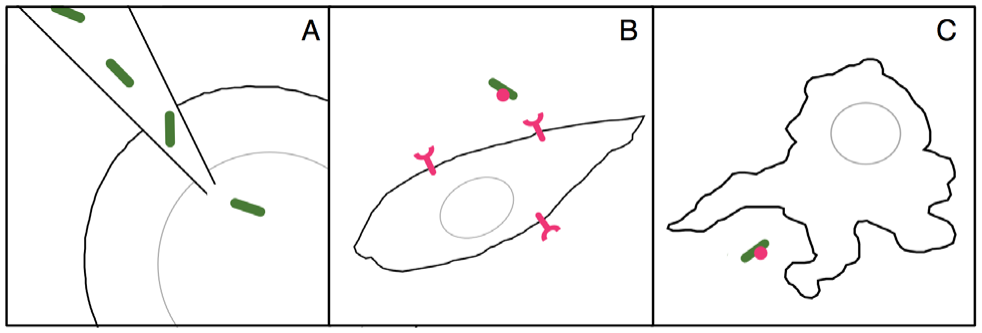
Three paths to endosymbiosis used in this study. A.) Direct microinjection of *S. elongatus* into zebrafish embryos allow exploration of *in vivo* dynamics of bacteria inside animal cells. B.) Invasion of mammalian cells through heterologous expression of invasin and listeriolysin O. C.) Phagocytosis of bacteria by macrophages. Bacteria subsequently escape from the endosomal compartment through expression of listeriolysin O.

There is a fine line between the pathological and beneficial in natural endosymbiotic events. Many endosymbiotic relationships that exist in nature are hypothesized to have begun through the acquisition of resistance to predation— bacterial resistance to lysosomal digestion by amoeba after phagocytosis or eukaryotic resistance to bacterial infection after intracellular invasion [17]. Replicating these events in the laboratory may lead to a partial endosymbiosis. Symbiosis is generally thought to refer to a mutualistic relationship where both partners benefit, but the term can be construed rather broadly; Lynn Margulis paraphrases de Bary's 1879 definition of symbiosis as simply the “protracted physical associations among organisms of different species, without respect to outcome.” [18] We explored three paths for entry of photosynthetic bacteria into animal cells that would satisfy this broad definition of symbiosis (figure 1)—direct microinjection into zebrafish embryos to explore the *in vivo* dynamics in a whole animal model, engineering with invasin from *Y. pestis* (inv) and listeriolysin O from *L. monocytogenes* (llo) to allow invasion of mammalian endothelial cells, and endocytosis of inv and llo engineered strains by macrophages. Invasin is a bacterial surface protein that interacts with mammalian β1-integrins and causes uptake of the bacterial cells, while listeriolysin O is a hemolysin that disrupts the endosomal membrane and allows bacteria to enter the mammalian cytoplasm post-uptake.

Invasive bacteria cause several deadly infectious diseases in humans, caused by intracellular pathogens such as *Y. pestis*, *L. monocytogenes*, and enteroinvasive *E. coli* [19]. Recent work in biological engineering and synthetic biology has focused on the development of non-infectious but invasive and deadly bacteria that target and destroy only specific cell types for disease treatment, particularly cancer [20], or for delivery of peptide [21] or nucleotide based vaccines [22] and RNA interference gene therapy [23].

Macrophages can take up and phagocytose many different species of bacteria. However, most species of bacteria, including many pathogens, are unable to replicate in the cytoplasm of mammalian cells, and the precise mechanism of growth inhibition is unknown and a matter of controversy [24]. In contrast, non-pathogenic *Bacillus subtilis* expressing heterologous hemolysin has been shown to escape phagosome digestion by macrophages and divide in the mammalian cytoplasm [25]. However, microinjection studies have found that only those species that naturally divide in the cytoplasm were able to replicate upon injection into mammalian cells, with even intravacuolar pathogens unable to divide in the cytoplasm [26]. To our knowledge, such experiments have not been attempted with photosynthetic bacteria or other autotrophs.

Nearly eighty years ago, photosynthetic algae were explored as symbionts for cells grown in tissue culture, as a method for renewing and replenishing growth media with oxygen and nutrients while removing waste products and carbon dioxide [27], [28]. More recently, photosynthetic symbiosis in tissue culture was explored as a method for understanding the nutritional requirements of host and symbiont [29]. We sought to explore the behavior of the photosynthetic bacteria *Synechococcus elongatus* inside eukaryotic cells as a platform for engineered photosynthetic endosymbiosis and found that cyanobacteria have little apparent effect on their host cells and can divide in the macrophage cytoplasm. Further engineering of metabolite production and secretion [30] in such endosymbiotic strains has the potential to lead to true mutualistic relationships between photosynthetic bacteria and mammalian cells, essentially creating artificial, engineerable, animal chloroplasts.

## Materials and Methods

### Cells and media

*E. coli* DH5α was used for all plasmid manipulation using standard procedures. *S. elongatus* PCC 7942 was cultured in BG-11 medium [31] at 30°C and illuminated by strong light. CHO and J774 cells were maintained using standard procedure in F-12 medium (Invitrogen) for CHO cells and RPMI 1640 medium (Invitrogen) for J774 cells. All media contained L-glutamine and were supplemented with 10% FBS (HyClone) and 1% Penicillin/Streptomycin Mix (Invitrogen). For culturing cells during infections outside of the controlled 5% CO2 atmosphere, Leibovitz's L-15 medium without phenol red (Invitrogen) was used for all cell lines, supplemented with 10% FBS for all cell types and 0.069 mg/ml proline for CHO cells.

### Plasmids and DNA construction

The invasin gene from *Yersinia pestis* (inv) was subcloned from the pAC-TetInv plasmid [16] provided by Chris Voigt (University of California, San Franscisco) and listeriolysin O (llo) was amplified from *Listeria monocytogenes* genomic DNA provided by Heather Kamp (Harvard Medical School, Boston MA). Invasin DNA was amplified with primers adding a SpeI site upstream (5′-CGCAACTAGTATGGTTTTCCAGCCAATCAG-3′) and NotI and XbaI sites downstream (5′-CTGCAGCGGCCGCTAGCTCTAGATTATATTGACAGCGCACAGA-3′) Listeriolysin was amplified with primers adding a SpeI site, a ribosome binding site, and a short spacer for cloning downstream of invasin (5′-CGCAACTAGT*AGGAGGAAAAACAT*ATGAAAAAAATAATGCTAGTTTT-3′) and NotI and XbaI site downstream (5′-CTGCAGCGGCCGCTTCTAGATTATTCGATTGGATTATCTA-3′). Invasin and listeriolysin were then sequentially subcloned into the pNS3 vector for homologous recombination into *Synechococcus* neutral site 3 [30].

The pNS3-invllo vector was incubated overnight in the dark with a culture of *S. elongatus PCC 7942* cells washed in 10 mM NaCl, and integration into the neutral site was selected using BG11 plates containing 1.5% Noble Agar and 12.5 µg/ml chloramphenicol. Expression of invasin and listeriolysin was induced with 100 µM IPTG for 24 hours.

### Zebrafish injection

Zebrafish embryos in the one-cell stage were injected with a solution containing mRNA for expression of membrane GFP (mGFP) and bacteria. Needles were pulled on a Sutter P2000 laser needle puller from Drummond glass capillary tubes. Eggs were injected with 2.3 nl of injection solution using a Nanoject. The injection solution consisted of injection buffer (50 mM NaCl, 1 mM Tris pH 8, 0.1 mM EDTA and 0.1% Phenol Red) and contained 40 ng of mGFP mRNA and 1 µl of a saturated bacterial suspension per 10 µl of injection solution. The bacterial suspension (*S. elongatus* or *E. coli*) was prepared by spinning down 1 ml of an overnight *E. coli* culture or a dense 24–48 h old, exponentially growing *S. elongatus* culture. The cells were resuspended in 1 ml of injection buffer without Phenol Red and again pelleted. The supernatant was removed; the cells were mixed and always used fresh for the injections.

Embryos were raised in egg water (0.3 g/L Instant Ocean, 75 mg/L CaSO_4_) slightly shaded from the light in the cyanobacterial incubator at 30°C. Egg water was changed as needed. To follow individual embryos over time, the embryos were separated from each other in 12-well plates.

Development of the embryos injected with bacteria was monitored with a fluorescence dissecting microscope. For confocal imaging the embryos were dechorionated and placed in imaging molds made from 1% (w/v) agarose in egg water. Mounted embryos were imaged in an upright Zeiss LSM 710 confocal microscope. The embryo was submerged in eggwater containing 1× Tricaine solution (10× Tricaine solution: 0.1% (w/v) Tricaine and 10 mM Tris in egg water adjusted to pH 7 with NaOH) for anesthetization.

All of our zebrafish protocols were approved by the Harvard Medical School (HMS) Office for Research Subject Protection and the HMS Standing Committee on Animals (IACUC Approval Number 04487).

### Mammalian cell invasion assay

For infections of mammalian cells with bacteria, induced bacteria were washed and transferred from their culture medium into PBS (137 mM NaCl, 2.7 mM KCl, 10 mM Na2HPO4, 2 mM KH2PO4). Bacterial suspensions in PBS were set to the same OD and 100 µl of this suspension were added per 2 ml of cell culture medium per well of 12-well tissue culture dishes containing the mammalian cells. L-15 medium during the infection did not contain antibiotics. After the treatment of the cells with *S. elongatus* for 3 hours to overnight, the cells were washed with PBS three times and the medium was replaced by L-15 containing 100 µg/ml gentamicin, an antibiotic that does not cross the mammalian cell membrane. During and after infections of the mammalian cells with bacteria, the cultures were kept at 30°C in atmospheric CO_2_ levels. For *S. elongatus*, cells were illuminated with fluorescent lamps from both sides of the tissue culture plate.

For time-course of *S. elongatus* infection in macrophages, 10,000 J774 cells were plated per well of a 96-well plate in L-15 media and were allowed to attach to the bottom overnight at 37°C in the dark. Following attachment, 10 microliters of wild type or inv/llo engineered *S. elongatus* diluted to OD_750_ of 0.025–0.4 in PBS (corresponding to approximately <1 bacteria per macrophage to >4 bacteria per macrophage) were added to each well and incubated at 30°C in the light for six hours. Each well was then washed in PBS and the media was replaced with L-15 containing 100 µg/ml gentamicin and plates were incubated at 30°C in the light. One plate was removed every 24 hours and cells were fixed in 3% paraformaldehyde, cells were permeablized in 0.01% Triton-X in PBS and stained with DAPI. Plates were stored at 4°C in the dark and imaged at the same time using fluorescence microscopy.

### FACS analysis of mammalian cells with intracellular bacteria

After 24 hour infection, CHO cells were washed in PBS, trypsinized, and resuspended in FACS buffer (PBS supplemented with 1%FBS). Cells were sorted with a BD FACSAria cell sorter based on red channel fluorescence. Cells positive for red fluorescence were gently reattached to glass-bottomed tissue culture dishes with concanavalin A and imaged with confocal microscopy.

## Results

### Zebrafish embryos injected with *S. elongatus* hatch and thrive

Photosynthetic symbiosis exists in several underwater species, such as the sea slug *Elysia chlorotica*, which incorporates the chloroplasts from algae that it feeds on into the cells of its intricately branched digestive system, allowing it to survive for months photoautotrophically [7]. While such a complex symbiotic relationship likely evolved over much longer time scales, we were interested in replicating the first step of an underwater photosynthetic symbiosis and exploring the *in vivo* dynamics of photosynthetic bacteria in a developing animal. We chose zebrafish embryos as they are easy to microinject, well studied, and are clear, allowing light to penetrate.

**Figure 2:**
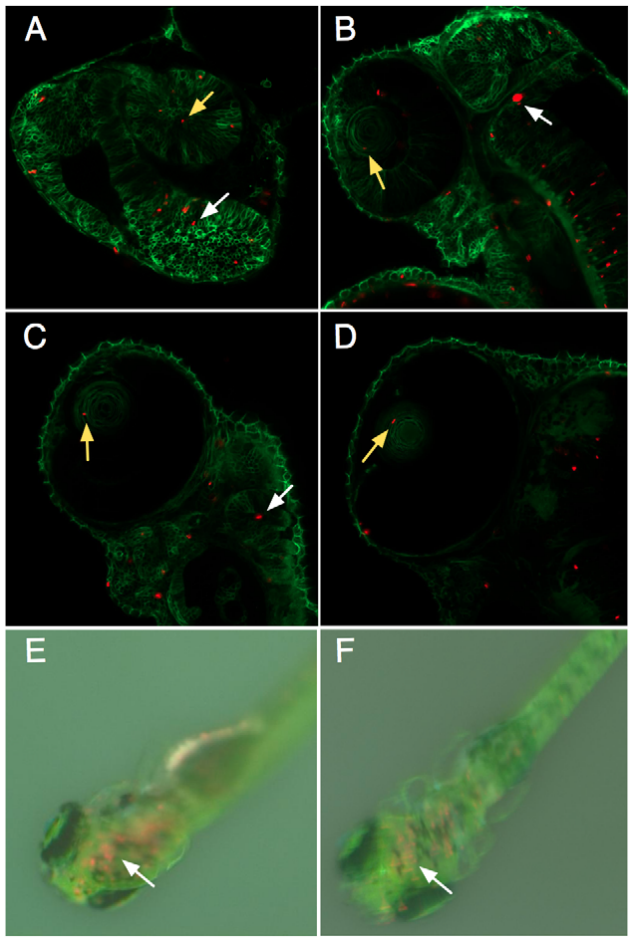
Tracking intracellular *S. elongatus* through zebrafish development. Single optical slice confocal microscopy images of the anterior of the zebrafish embryo at A.) Day 1 post injection, B.) Day 2, C.) Day 3, D.) Day 4, and dissecting microscope images of embryos E.) Day 8, F.) Day 12 post injection. Zebrafish cell membranes are outlined in green, with red autofluorescent bacteria visible in cells throughout the embryo, including the eye (yellow arrows) and brain (white arrows). Red autofluorescence gradually decreased over the course of experimental observations, but remained visible in the brain of the young zebrafish even after 12 days.

Up to ten million bacteria were injected into zebrafish embryos at the single cell stage to track the relationship between the vertebrate and bacterial cells through development. Red autofluorescent bacteria were found intracellularly throughout the embryo during development, including in the brain and even the lens of the eye (figure 2A–D) with no discernible morphological effects. *Synechococcus* survived inside the embryo's cells for up to twelve days based on continued red autofluorescence (figure 2F), at which time the experiment was terminated as the fish began to develop pigment that would block light to the intracellular bacteria.

**Figure 3:**
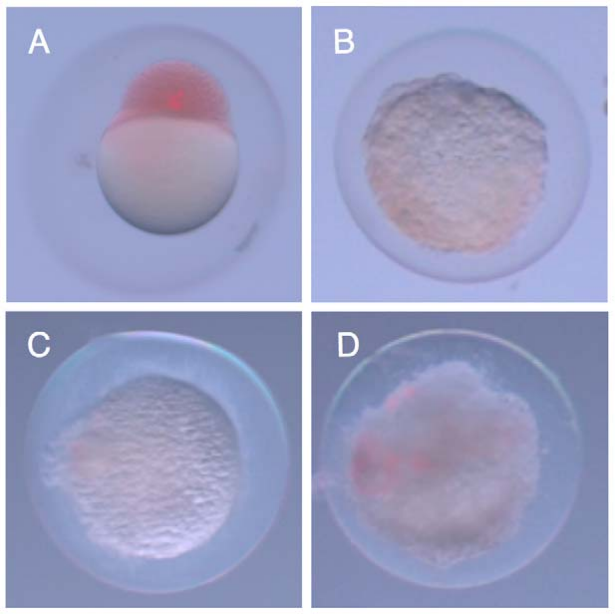
Zebrafish embryos are immediately killed by *E. coli*. A.) Zebrafish embryo two hours after injection of *S. elongatus*. Cells appear red due to phenol red present in the injection buffer. B.) Injection of *E. coli* led to drastic morphological changes in the embryo after two hours, and this change was observed with *E. coli* cells that were C.) UV killed, or D.) ΔmsbB mutants.

In stark contrast, injecting *E. coli* cells killed the embryo within two hours (figure 3B). This rapid death occurred even when the *E. coli* were UV killed prior to injection (figure 3C) and when the Lipid A production was attenuated by deletion of msbB (figure 3D), a modification shown to decrease incidence of septic shock by tumor-targeting *Salmonella* [32]. These data point to other surface markers that can cause the death seen in the *E. coli* injected embryos and a benign role for the non-pathogenic *S. elongatus in vivo*.

### *Synechococcus* expressing invasin and listeriolysin invade mammalian cells

We also sought to explore a more physiological model of intracellular invasion than direct microinjection. The bacterial virulence factors encoding mammalian cell invasion—invasin from *Yersinia pestis* [33]—and escape from the lysosomal compartment—listeriolysin O from *Listeria monocytogenes* [34] have been identified, cloned, and shown to confer invasive behavior to non-pathogenic bacterial species. We inserted invasin and listeriolysin as a tandem operon in *S. elongatus* neutral site 3 [30] and incubated induced *S. elongatus* cells with CHO cells at 50%–80% confluence overnight at 30°C in bright light.

**Figure 4:**
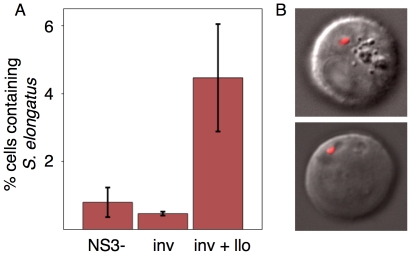
Invasion of CHO cells. A.) *S. elongatus* engineered with invasin and listeriolysin are able to invade CHO cells at a higher efficiency than *S. elongatus* harboring the empty vector or invasin alone. Cells positive for red fluorescence were sorted by FACS and B.) observed under confocal microscopy, showing intracellular localization of at least one bacterial cell per CHO cell in the majority of the cells observed.

While expression of invasin alone is sufficient for high efficiency invasion by *E. coli* for multiple mammalian cell types in culture (HeLa, U2OS, HepG2, CHO [16]), expression of both invasin and listeriolysin was required for invasion of CHO cells by *S. elongatus*, a result previously reported for engineered *E. coli* cells invading colon cancer cells [23]. The invasion efficiency was such that 4.8% of mammalian cells were positive for the red channel autofluorescence from intracellular photosynthetic bacteria as measured by fluorescence-activated cell sorting (FACS) analysis (figure 4A). Sorted cells were imaged with confocal microscopy (figure 4B), confirming intracellular localization with approximately one bacterial cell per CHO cell analyzed.

### Replication inside mammalian macrophages

**Figure 5:**
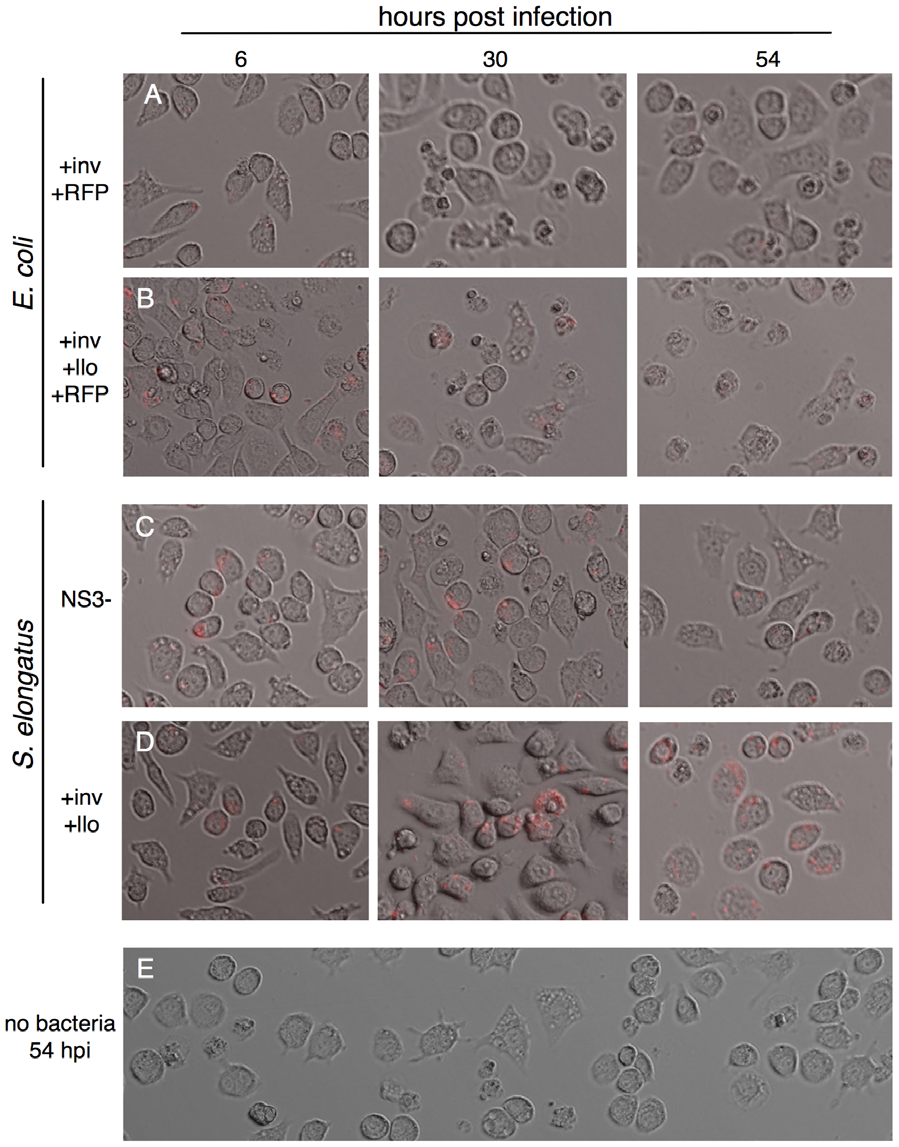
*E. coli* and *S. elongatus* lead to differential effects when phagocytosed by macrophages. Large scale granulation is observed when macrophages take up *E. coli* that is A.) not expressing llo and to an even greater extent with B.) *E. coli* expressing llo off of the inducible lac promoter of the pNS3 vector. In contrast, macrophages displayed similar morphology two days after infection with C.) empty vector *S. elongatus*, D.) *S. elongatus* expressing inv and llo, and E.) macrophages untreated with bacteria but maintained at 30°C in bright light for two days.

Bacteria can also enter cells through phagocytosis, and escaping digestion by the lysosome is a prerequisite for pathogenic and symbiotic growth. Macrophages are a crucial part of the mammalian immune system, seeking out, engulfing and digesting foreign bodies and bacteria. The immortal mouse macrophage cell line J774 will quickly engulf large numbers of bacterial cells in culture. We therefore incubated plates of 50% confluent macrophages with varying concentrations of bacterial cells for one hour at 37°C for *E. coli* and six hours at 30°C for *S. elongatus*. As with zebrafish embryos, engulfed *E. coli* cells will quickly kill their host macrophages (figure 5A), while even high numbers of *S. elongatus* cells will remain inside J774 for several days with relatively little effect.

*E. coli* highly expressing listeriolysin were observed to kill macrophages faster than wild type *E. coli* (figure 5B). However, after two days of incubation with *Synechococcus*, similar levels of cell death were observed in macrophages with *Synechococcus* with only the empty vector integrated (figure 5C), those expressing invasin and listeriolysin (figure 5D), and with macrophages without any bacteria (figure 5E).

Non-pathogenic bacteria that have been engineered with listeriolysin O to escape the macrophage endosome have been shown to replicate in the cytoplasm [25]. However, there are many factors in the mammalian cytoplasm speculated to be involved in preventing bacterial growth, a fact suggested by the extremely small number of intracellular pathogens able to divide in the presumably nutrient-rich cytoplasm [24]. *S. elongatus* requires little external metabolic input and grows at a relatively fast rate at intracellular carbon dioxide concentrations (one division every 8–12 hours). In addition, as we have shown (above) *S. elongatus* has a special relationship to eukaryotic antimicrobial systems as it is able to peacefully coexist with animal cells. As such, it is expected that *S. elongatus* engineered with listeriolysin O will be able to divide in the mammalian cytoplasm.

**Figure 6:**
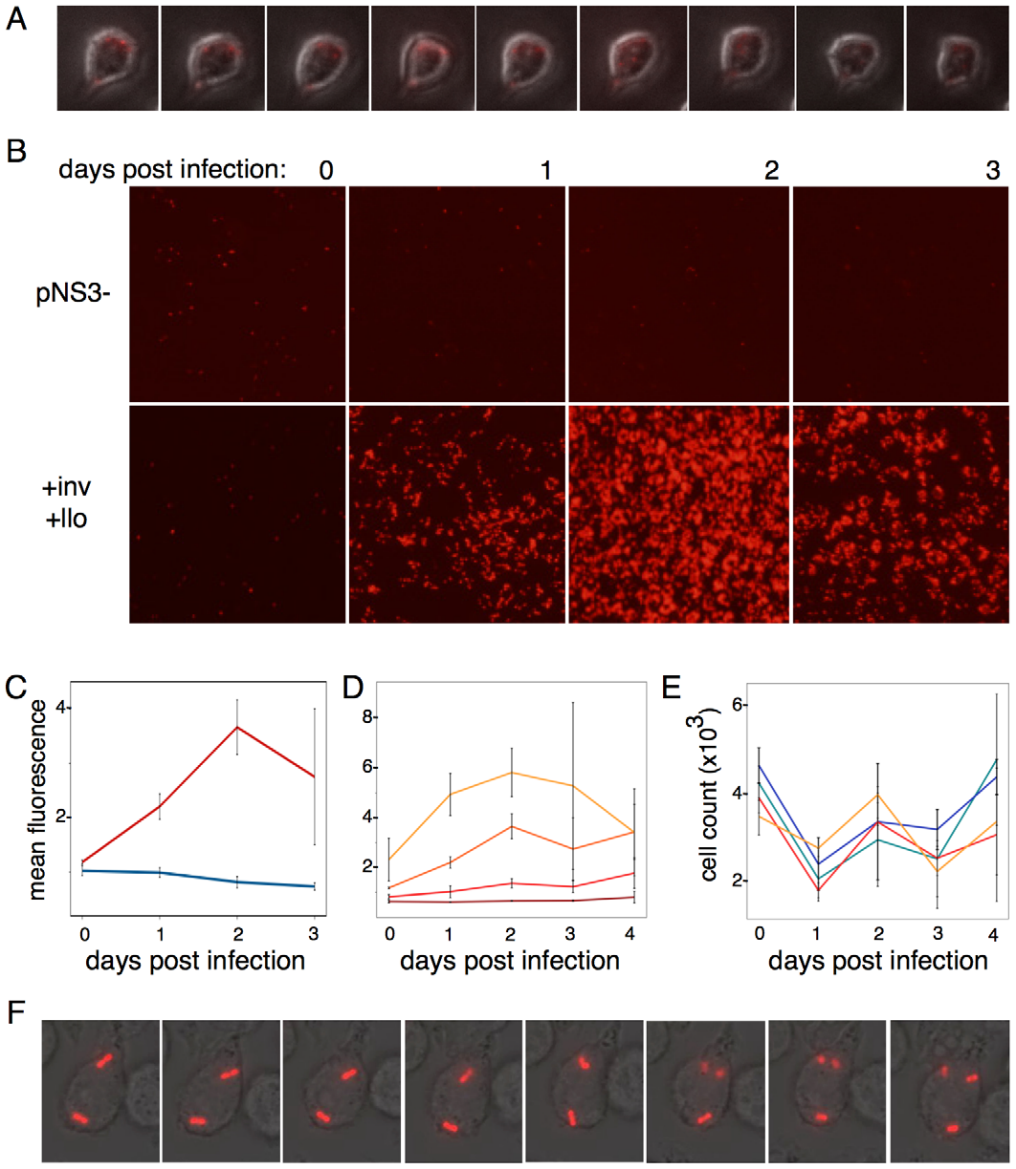
*S. elongatus* can grow inside the macrophage cytoplasm. A.) Time lapse microscopy of macrophages infected with +inv+llo *S. elongatus* kept in the dark shows the gradual decrease in red autofluorescence over the course of 12 hours. In contrast, when kept in the light, B.) empty vector *S. elongatus* autofluorescence is observed to gradually decrease over the course of several days (top row), while a significant increase in red *S. elongatus* autofluorescence was observed in macrophages infected with inv llo *S. elongatus* for two days post-infection (bottom row). This fluorescence was observed to decrease after the third day of infection. C.) This change in fluorescence over time can be quantified as a change in background subtracted mean fluorescence in ImageJ and averaged over triplicate experiments. Empty vector (blue line) and +inv+llo *S. elongatus* (red line) show marked differences in growth when infected at similar densities of 1–2 bacterial cells per macrophage. D.) +inv+llo *S. elongatus* displayed infection density dependent growth rates in macrophages. Each line shows change in mean fluorescence in cells infected at a single starting density, ranging in multiples of two from fewer than one cell per macrophage to approximately 4 bacteria per macrophage. E.) Macrophage cell counts were variable across replicates and over the course of the experiment but displayed no significant difference between macrophages infected with empty vector *S. elongatus* at low (green line) or high density (blue line), or +inv+llo *S. elongatus* at low (red line) or high (yellow line) density. F.) When infected at low density of fewer than one bacteria per macrophage, *S. elongatus* division was observed during 18 hour time-lapse fluorescent microscopy in approximately 1% of macrophages observed, in particular those cells that contained more than one bacterial cell due to stochastic fluctuations.

In the dark, *S. elongatus* phogocytosed by macrophages will rapidly lose red channel autofluorescence over the course of 12 hours, indicating death (figure 6A). In the light, wild type *S. elongatus* autofluorescence will more slowly decrease in intensity over several days (figure 6B, top row). *S. elongatus* engineered with invasin and listeriolysin, able to escape lysosome digestion, showed marked increase in autofluorescence in the first two days post-infection, with the number of autofluorescent bacteria decreasing only after 3 days (figure 6B, bottom row).

The rate of division in the macrophage cytoplasm was quantified for varying densities of *S. elongatus* in 96 well plates. Mean, background subtracted fluorescence was averaged for triplicate infections. At similar starting density of approximately 2 bacteria per macrophage, there is marked contrast between empty vector (blue) and +inv+llo (red) *S. elongatus* (figure 6C). Rates of division in the engineered strain were correlated to *S. elongatus* infection densities. At the lowest concentrations, with fewer than one bacterial cell per macrophage, the engineered strain is digested more slowly than wild type *S. elongatus*, but does not show large-scale evidence of division, but as infection density is doubled, the rate of growth increases and begins to level off at the highest density (1–4 bacteria per macrophage, figure 6D). Differences in *S. elongatus* growth did not correlate with decrease in macrophage cell counts over time, which remained variable but consistent between wells at different infection densities over time (figure 6E). Even at the lowest infection density, +inv+llo *S. elongatus* division was observed in approximately 1% of cells tracked with time-lapse microscopy (figure 6F).

## Discussion

Complex relationships between many different species of organisms characterize the biological world, but the details of these symbiotic relationships have proven difficult to untangle through reductionist experimentation. Simplified, engineered multi-species relationships can provide a framework for studying natural symbiotic relationships [35]. We show that photosynthetic bacteria can be engineered to invade and divide inside mammalian cells for use as a platform for further engineering or study of evolutionary dynamics of endosymbiosis.

A synthetic approach to photosynthetic mutualism in animal tissue culture has been attempted previously many years ago, with mixed populations of algae and animal cells showing gas and nutrient exchange in culture [27], [28], [36]. More recently, amoeba infected with a naturally occurring parasitic bacteria and carefully cultivated over several years eventually became dependent on the bacterial symbiont, showing that endosymbiosis can be established quite rapidly under the right conditions [37].

Natural endosymbiosis between photosynthetic organisms and animal species occurs in many marine species such as corals and sponges, whose simple body plans and high surface-to-volume ratios make such associations valuable [38]. While these marine photosymbioses have been studied for many years, the first evidence of a facultative photosynthetic endosymbiosis in vertebrates was only recently discovered between the embryo of the spotted salamander and green algae [39]. Algal-salamander associations had previously been observed extracellularly [40], with gas exchange between the algae and salamander shown to be beneficial but not required for the developing embryo [41]. These newly discovered intracellular interactions occur only in the embryo, with algae dying by the time the larvae begins to feed and no evidence of vertical transmission from the underground-dwelling adult salamander.

Such natural events show how rare bacterial-vertebrate endosymbiosis is, as well as how benign photosynthetic associations can be when they are established. We used a synthetic approach to developing photosynthetic associations with animal cells, finding that injecting *S. elongatus* into the zebrafish embryo does not affect fish development. As in the natural endosymbiosis, the photosynthetic cells slowly died, but remained in the animal for several weeks.

There are no known mammalian endosymbioses, and the mammalian cytoplasmic environment also remains poorly studied, with little understood about virulence factors that promote pathogenic intracellular growth in the handful of bacterial species able to replicate in the mammalian cytoplasm or mammalian factors that prevent growth and target bacterial pathogens for destruction [24]. Furthermore, while our understanding of microbial metabolism in isolated pure cultures is deep for commonly studied organisms, the metabolism of bacterial communities is currently probed primarily through metagenomics techniques that remain limited [42]. The metabolism of symbiotic bacteria or pathogens living *in vivo* is likewise poorly characterized, with evidence that *in vivo* metabolism differs significantly from that in pure laboratory culture [43].

Intracellular pathogens and symbionts alike must be able to take nutrients from their cellular hosts. Genes such as Hpt, the glucose-6-phosphate translocase from *L. monocytogenes*, allow for assimilation of host sugars [44]. Studies with auxotrophic strains of *L. monocytogenes* show the extent of the external metabolic requirement for intracellular division [43], [45], [46]. In contrast, *S. elongatus* requires very little input from its external environment besides light, carbon dioxide, and a small number of salts and minerals [31]. This minimal requirement may be central to our observation that photosynthetic bacteria can replicate inside the macrophage. Indeed, non-pathogenic photosynthetic autotrophs seem to have a privileged relationship with eukaryotic cells, able to coexist and even grow inside with relatively little damage to the host cell compared to even non-virulent *E. coli*.

Engineering a mutualistic metabolic endosymbiosis remains extremely difficult due to the sheer metabolic requirement of immortalized cells in culture. Based on concentrations of glucose and fructose secreted by engineered strains of *S. elongatus* [30] we estimate that each CHO cell would require on the order of 25 cyanobacterial cells to sustain growth, and J774 macrophages would require approximately 14,000 bacteria per cell to provide adequate glucose supply, numbers significantly higher than those observed in our experimental analysis. Relationships based on other secreted metabolites, small molecules, or enzymes may prove to be adequate for establishing an engineered mutualism. Additionally, improvements in the secretion of essential nutrients by *Synechococcus* will further aid this approach.

Just as synthetic biology can be used to query the principles underlying complex signaling or transcriptional networks, a synthetic approach can be used to uncover the complex dynamics underlying symbiotic relationships. Engineering of microbial communities and (endo)symbioses between different species has tremendous potential as a tool for synthetic biology [47], where growth is limited by the complexity of combining modular genetic devices in a cellular context [48]. Communities of cells working together can achieve results that pure cultures cannot. An engineerable photosynthetic symbiont can provide a light-controlled, orthogonal platform for engineering animal cells.
